# BabaoDan attenuates high-fat diet-induced non-alcoholic fatty liver disease via activation of AMPK signaling

**DOI:** 10.1186/s13578-019-0339-2

**Published:** 2019-09-18

**Authors:** Dandan Sheng, Shanmin Zhao, Lu Gao, Huifei Zheng, Wenting Liu, Jing Hou, Yuxiang Jin, Fei Ye, Qiudong Zhao, Rong Li, Naping Zhao, Li Zhang, Zhipeng Han, Lixin Wei

**Affiliations:** 1 Tumor Immunology and Gene Therapy Center, Third Affiliated Hospital of Second Military Medical University, NO. 225 Changhai Road, Shanghai, 200438 China; 2 Department of Pharmacy, Changhai Hospital, Second Military Medical University, Shanghai, 200433 China

**Keywords:** Babaodan, Non-alcoholic fatty liver disease, Lipid metabolism, AMPK pathway

## Abstract

**Background:**

Babaodan (BBD), a traditional Chinese medicine, has been shown to have protective effects during liver injury and ameliorate liver disease progression, but little is known about its effect on non-alcoholic fatty liver disease (NAFLD). The aim of this study was to investigate the effects of BBD on obesity-induced NAFLD.

**Methods:**

C57BL/6 J mice were fed with normal diet, high fat diet (HFD) or HFD + BBD for 8 weeks. Weights of all mice were recorded every 3 days. At the end of the experiments, the level of livers, kidneys and adipose tissues of each animal was weighed. Blood serum levels of alanine aminotransferase (ALT), aspartate aminotransferase (AST), total cholesterol (TC), triglyceride (TG), high density lipoprotein cholesterol (HDL-C) cholesterol, low density lipoprotein cholesterol (LDL-C), glucose and leptin were detected with appropriate test kits. Haematoxylin–eosin (HE), Masson trichrome and Oil Red O staining of the liver were performed. We applied immunohistochemical analysis to investigate the expression of TNF-α, IL-6 and leptin in liver tissue. The expression of genes related lipid anabolism (SREBP1-c, ACC, SCD-1, LXRα and CD36) and ß-oxidation (CPT-1 and PPARα) in liver and adipose tissues was determined by RT-PCR. The expression of AMPK and p-AMPK was determined by western blot analysis.

**Results:**

We found the weight of bodies and tissues (retroperitoneal fat pads, kidneys and livers) of mice fed with HFD + BBD were significantly lower than that of HFD-fed mice. And liver injury induced by HFD was relieved in mice treated with BBD, accompanied with significant reduction were observed in serum ALT/AST activities and alleviated pathological damage. The levels of glucose, TG, TC, HDL-C and LDL-C in the liver or serum were significantly decreased on HFD + BBD group compared with HFD group. Furthermore, BBD treatment reduced the level of TNF-α and IL-6 induced by HFD. The level of leptin in the liver and serum were reduced in mice fed with HFD + BBD than that of HFD-fed mice. Several lipid synthesis genes (SREBP1-c, ACC, SCD-1, LXRα and CD36) were down-regulated and that of ß-oxidation (CPT-1 and PPARα) up-regulated in HFD + BBD group compared with HFD group. In addition, BBD increased the expression of p-AMPK compared with untreated HFD group, which suggested BBD improved the activation of AMPK pathway.

**Conclusion:**

In summary, our results indicate that BBD has potential applications in the prevention and treatment of NAFLD, which may be closely related to its effect on lipid metabolism via activation of AMPK signaling.

## Background

Obesity is defined as an excessive body weight followed by accumulated adipose tissue [1]. A recent study showed that about 108 million children and 604 million adults are obese, and these numbers are on the rise [2]. Obesity is becoming one of the most serious public health problems worldwide. Moreover, obesity individuals have a high risk of numerous diseases, such as non-alcoholic fatty liver disease (NAFLD), a major chronic liver disease, which could lead to liver cirrhosis, liver cancer, and ultimately death [3]. NAFLD is frequently associated with increased visceral adiposity and metabolic abnormalities. A recent metaanalysis involving over 8.5 million individuals from 22 countries showed that more than 80% of NAFLD patients were overweight or obese, 72% had dyslipidemia [1, 4, 5]. The prevalence of NAFLD has increased in many countries. Approximately 25% of adults in the United States have fatty liver in the absence of excessive ethanol consumption [3]. In China, fatty liver disease is increasing at a rate of 0.594% per year and is expected to afflict 20% of Chinese by 2020 [6]. Therefore, NAFLD is becoming the most common liver disease worldwide. Therefore, great efforts have been continuously put into developing new therapeutic agents for obesity and NAFLD.

Traditional Chinese medicine (TCM) with a long history of clinical practices plays a crucial rule in disease treatment. For instance, Babaodan (BBD) as a classical TCM, has been showed to exert curative function in liver damage [7]. BBD has also been proved to be an adjuvant therapeutic strategy combining with chemotherapy in liver cancer, gastric cancer and lung cancer treatment [7]. Recently our laboratory has demonstrated that BBD ameliorated hepatic fibrosis through inhibiting activation of hepatic stellate cell and further protected liver from injury [8]. However, there is little evidence about BBD function exerted in obesity and associated diseases. In present study, we evaluated the effects of BBD on high fat diet-induced NAFLD.

## Methods

### Animals

Male C57BL/6 mice aged 6 weeks were purchased from the Shanghai Experimental Animal Center of the Chinese Academy of Sciences (Shanghai, China) and housed in maintained at the laboratory animal center of the Second Military Medical University. Experiments and procedures were approved by the Animal Ethics Committee of the Second Military Medical University. All animal handling and study procedures complied with the current Chinese regulation, GB14925-2010: Laboratory animal requirements of environment and housing facilities (Chinese version).

### Experimental design

BBD was a gift from Shanghai Pharmaceutical Group Inc. All animals were acclimatized on a normal diet for 1 week and then divided into 3 study groups (7 mice/group): (1) control group fed normal diet (ND, composed of 12%-fat, 23%-protein, 65%-carbohydrates based on caloric content), (2) group fed high fat diet (HFD, composed of 40%-fat, 20%-protein, 40%-carbohydrates based on caloric content), (3) group fed HFD + BBD (25 mg/kg) and experimental period was 8 weeks. BBD was administered every 3 days by oral gavage for 8 weeks. At the end of feeding period, peripheral blood, liver, kidney and retroperitoneal fat pads were collected.

### Body and tissue weight

Individual animals were weighed from the first BBD treatment every 3 days. The livers, kidneys and retroperitoneal fat pads of each animal were removed and weighed after the feeding experiment.

### Biochemical and ELISA analysis

The concentrations of total cholesterol (TC), triglyceride (TG), high density lipoprotein cholesterol (HDL-C), cholesterol, low density lipoprotein cholesterol (LDL-C), alanine aminotransferase (ALT), aspartate aminotransferase (AST) and glucose in serum were tested with a biochemical auto-analyzer (Fuji Medical System, Tokyo, Japan) according to the manufacturer’s instructions. Tumor necrosis factor α (TNF-α), Interleukin 6 (IL-6) and leptin were measured with kits obtained from Abcam.

### Histopathological analysis

The tissue specimens were embedded in paraffin wax, sliced to 4 μm thickness, and placed on separate glass slides. After hematoxylin–eosin (HE) staining, Oil Red O staining and Masson trichrome staining, respectively, the sections were observed by light microscope.

### Immunohistochemical analysis

For immunohistochemistry staining of TNF-α, IL-6 and leptin in liver tissue sections were deparaffinized in xylene and dehydrated with graded ethanol. After washing with dH_2_O, tissue peroxidase was blocked with 3.0% hydrogen peroxide in methanol for 20 min at room temperature. For antigen retrieval using citric acid buffer, the slides were heated at 120 °C for 20 min and then cooled for 20 min at room temperature. After incubated overnight with the primary antibody IL-6 (1:800, Abcam, UK), TNFα (1:200, Abcam, UK) and leptin (1:500, Abcam, UK), and then with peroxidase-conjugated streptavidin for 15 min. Diaminobenzidine–tetrahydrochloride (DAB) was used as the substrate to detect antigen–antibody binding. Sections were counterstained with hematoxylin and observed under the microscope.

### Real time-PCR

To test mRNA expression, total RNA from every frozen mouse livers tissue was extracted by using TRIZOL reagent (Invitrogen, Carlsbad, CA, USA). Prime Script RT reagent Kit (Takara, Kyoto, Japan) was performed for cDNA synthesis. Relative quantitative PCR (RT-PCR) was performed using SYBR Green PCR Kit (Applied Biosystems, Foster City, CA, USA) according to the manufacturer instructions. The level of mRNA was expressed as the ratio of the signal intensity for each gene relative to that of GAPDH. Primer sequences are listed in Table 1.

**Table 1:** Primer sequence for real-time polymerase chain reaction

Gene	Primer sequence
ACC	
F	CCGTTGGCCAAAACTCTGGAGCTAA
R	GAGCTGACGGAGGCTGGTGACA
LXR-α	
F	CTCAATGCCTGATGTTTCTCCT
R	TCCAACCCTATCCCTAAAGCAA
SCD-1	
F	CGGTCATCCCATCGCCTGCTCT
R	GTAGGCGAGTGGCGGAACTGC
SREBP1-c	
F	GTGAGGCGGCTCTGGAACAGAC
R	ATAGGGGGCGTCAAACAGGCC
CD36	
F	CACAGCTGCCTTCTGAAATGTGTGG
R	TTTCTACGTGGCCCGGTTCTAATTC
PPARα	
F	ACTGGTAGTCTGCAAAACCAAA
R	AGAGCCCCATCTGTCCTCTC
CPT-1	
F	TCTAGGCAATGCCGTTCAC
R	GAGCACATGGGCACCATAC
GAPDH	
F	ACCCCAGCAAGGACACTGAGCAAG
R	GGCCCCTCCTGTTATTATTATGGGGGT

### Western blot analysis

The fresh liver tissue was washed with PBS and lysed by RIPA and PMSF at a ratio of 100:1. The insoluble material was removed by centrifugation (12,000*g*) for 20 min at 4 °C. The protein concentration in the supernatant was determined by BCA Protein Assay Kit (Beyotime, Jiangsu, China). Equal amount of proteins was separated by SDS-PAGE and western blot analysis. The glyceraldeyde-3-phosphate dehydrogenase (GAPDH) antibodies were obtained from Bioworld Technology (AP0063). The total AMP activated protein (AMPK) (5832 s), phospho-AMPK (p-AMPK) (2535S), antibodies were obtained from Cell Signaling Technology (CST, Boston, USA).

### Statistical analysis

Results are presented as mean ± SD. Statistical differences between groups were analyzed by the Student’s *t* test. Statistical tests of data were performed using the GraphPad Prism6 software (San Diego, CA, USA). *p *< 0.05 was considered statistically significant.

## Result

### BBD ameliorates lipid accumulation of HFD-induced obesity mice

**Fig. 1:**
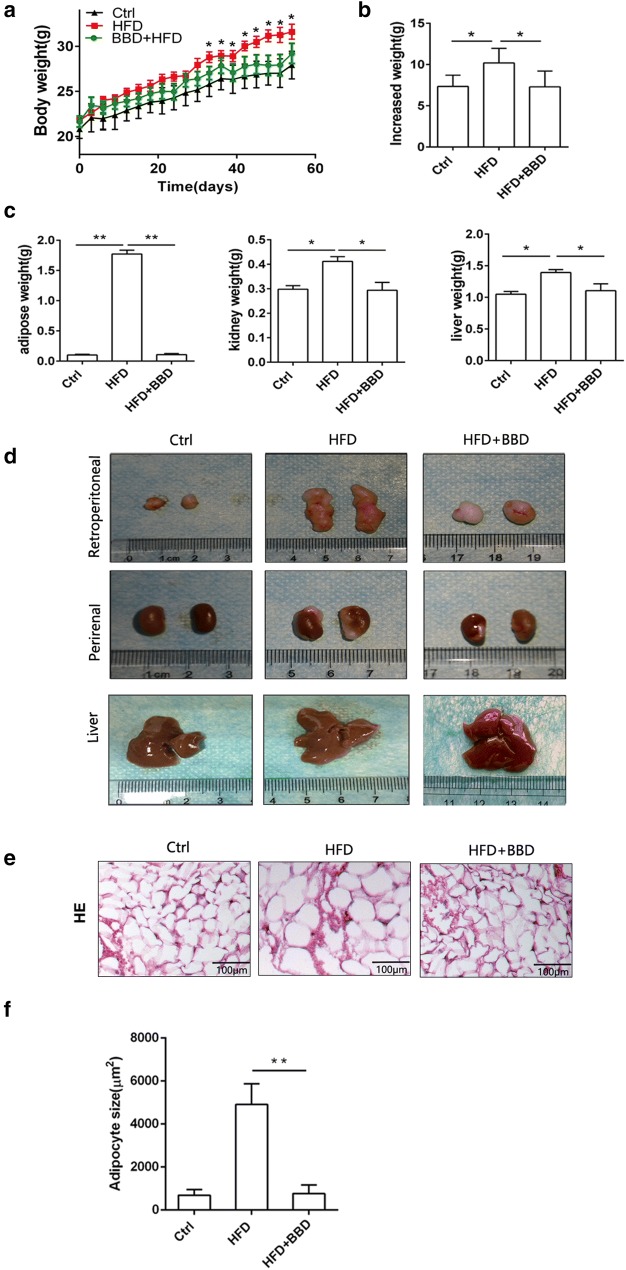
BBD ameliorates lipid accumulation of HFD-induced obesity mice. **a** Changes in body weight in C57BL/6 mice fed normal diet (n = 7), HFD (n = 7) or HFD + BBD (n = 7) (HFD + BBD-fed mice *p < 0.05 vs. HFD-fed mice). **b** Body weight gain of different groups. **c** Weight of retroperitoneal fat pads, kidneys and livers in mice of different groups. **d** Representative pictures of retroperitoneal fat pads, kidneys and livers from different groups. **e** The cell sizes of the epididymal adipose tissues for HE-staining are shown. **f** Quantification of adipocyte size of different groups. Error bars reflect SD, *p < 0.05, **p < 0.01

In order to investigate BBD effects on obesity, we conducted the model of obesity mice with HFD for 8 weeks. We found the weight of HFD mice was higher than that of normal diet group (Fig. 1a). Administration of BBD significantly decreased the weight gain compared to the high-fat group from the seventh week (Fig. 1a). The weight gained in the HFD + BBD group was significantly lower than that of HFD group at the end of feeding period (Fig. 1b). Meanwhile, retroperitoneal fat pads, kidneys and livers of mice fed with HFD + BBD were significantly lower than that of HFD-fed mice (Fig. 1c). We observed HFD-fed mice have more fat in retroperitoneal fat pads and perirenal fats compared with normal diet group and HFD + BBD group (Fig. 1d). The liver color of the high-fat control group appeared light yellow and that the normal group was dark red, while the liver color of HFD + BBD changed dark red (Fig. 1d). Furthermore, the epidydimal adipocyte size in HFD + BBD group was also lower compared with the HFD group (Fig. 1e, f). Taken together, these results demonstrate that BBD administration effectively prevents fat accumulation.

### BBD relieves liver injury induced by high fat diet

**Fig. 2:**
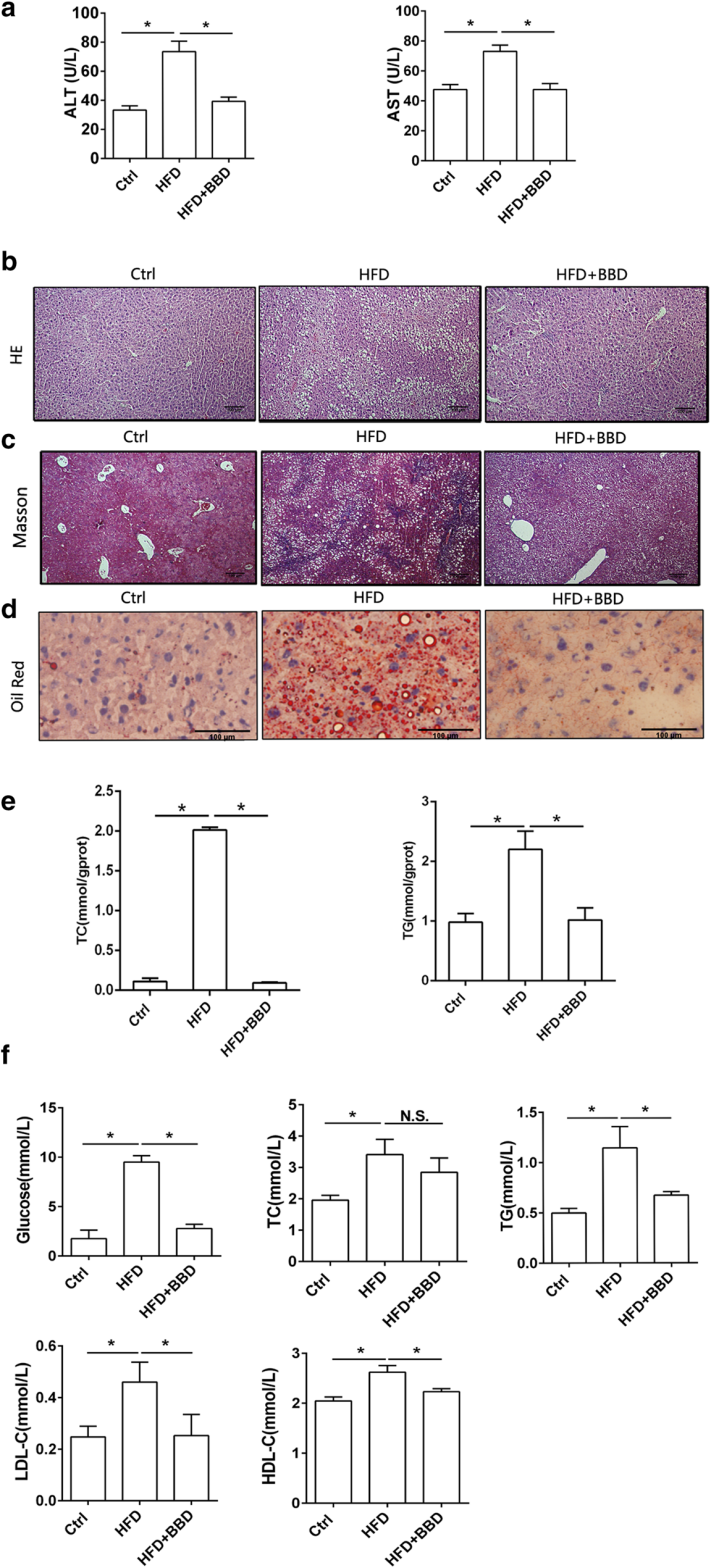
BBD relieves liver injury induced by HFD. **a** Activity of ALT and AST in serum of mice fed normal diet, HFD or HFD + BBD; Representative images of **b** H&E, **c** Masson’ trichrome and **d** oil-red O staining of the liver sections of different groups. **e** Relative cholesterol (TC) and triglyceride (TG) level in livers of different groups. **f** The level of glucose, total cholesterol (TC), low-density lipoprotein cholesterol (LDL-C), triglyceride (TG), high-density lipoprotein cholesterol (HDL-C) in serum of different groups. The data were shown as mean ± SD. n  =  7 for all groups. *p < 0.05

We detected HFD leaded to obvious liver injury in mice, as indicated by elevation of serum ALT and AST activities (Fig. 2a) and severe pathological damage (Fig. 2b, c). It was showed that hepatic fat accumulation in HFD group was obviously more than that in normal diet group. This suggested that long-term consumption of a high fat diet leads to the development of nonalcoholic fatty liver disease (NAFLD) in mice. To examine whether BBD affected liver injury induced by HFD, liver function indicators, morphological changes and the lipid contents in the liver of mice fed with HFD + BBD was detected. Histopathological analyses showed that BBD treatment relieved liver injury induced by HFD, accompanied with significant reduction in serum ALT/AST activities (Fig. 2a) and alleviated pathological damage (Fig. 2b). The HFD-group exhibited extensive liver bridging fibrosis and substantial collagen deposition (Fig. 2c). However, the HFD + BBD group had less bridging fibrosis and collagen (Fig. 2c). Meanwhile, BBD ameliorated the hepatic lipid droplets compared with no-treatment HFD mice (Fig. 2d). Additionally, BBD decreased the level of cholesterol and triglyceride in livers (Fig. 2e). As shown in Fig. 2f, the serum levels of glucose, TG, HDL-C and LDL-C were significantly decreased in the HFD + BBD group compared with the HFD group. And the serum TC of the HFD + BBD group was lower than that of the HFD group, although the difference was not statistically significant (Fig. 2e). Taken together, these results clearly demonstrate that BBD administration effectively relieve liver injury induced by high-fat diet through preventing hepatic fat accumulation.

### BBD relieves liver inflammation induced by high fat diet

**Fig. 3:**
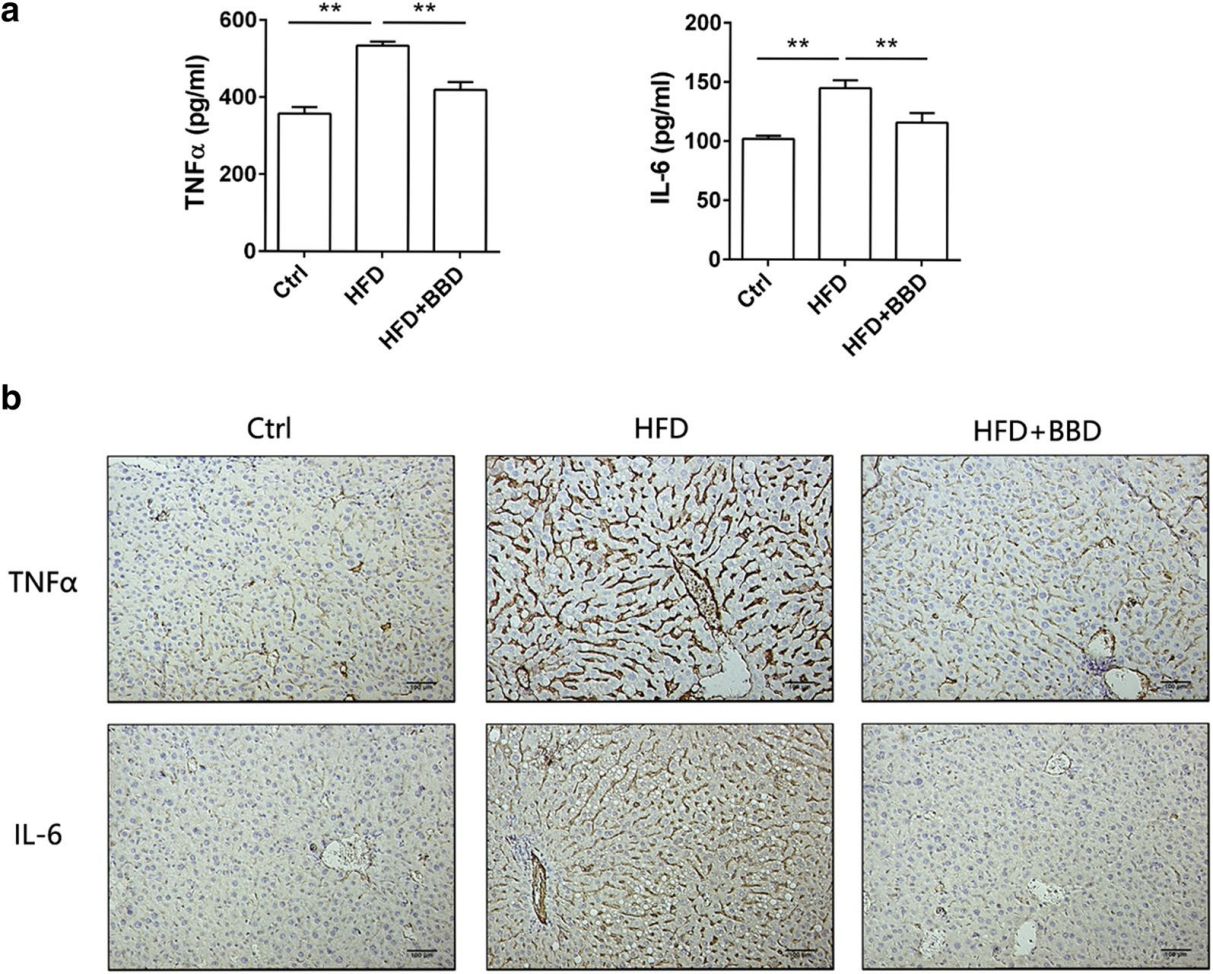
BBD inhibits liver inflammation induced by HFD. **a** The concentration of TNF-α and IL-6 in serum of mice fed normal diet, HFD or HFD + BBD; **b** representative immunohistochemistry staining for TNF-α and IL-6 in liver tissues. The data were shown as mean ± SD. n  =  7 for all groups. **p < 0.01

As obesity is a chronic inflammation that could cause damage to the liver, the liver inflammation was examined in each group. The expression of tumor necrosis factor α (TNF-α) and Interleukin-6 (IL-6) were detected to access the role of BBD in inhibition of inflammation. As Fig. 3a showed, the serum levels of TNF-α and IL-6 were elevated in HFD group compared with normal diet group. BBD treatment reduced the serum levels of TNF-α and IL-6 induced by HFD (Fig. 3a). Additionally, the expression of TNF-α and IL-6 in liver analyzed by immunohistochemically staining also demonstrate that the inflammation induced by high fat diet were reduced by BBD (Fig. 3b). In conclusion, BBD ameliorated obesity-induced liver inflammation.

### BBD activates AMPK pathway to suppress expression of hepatic genes involved in lipogenesis

**Fig. 4:**
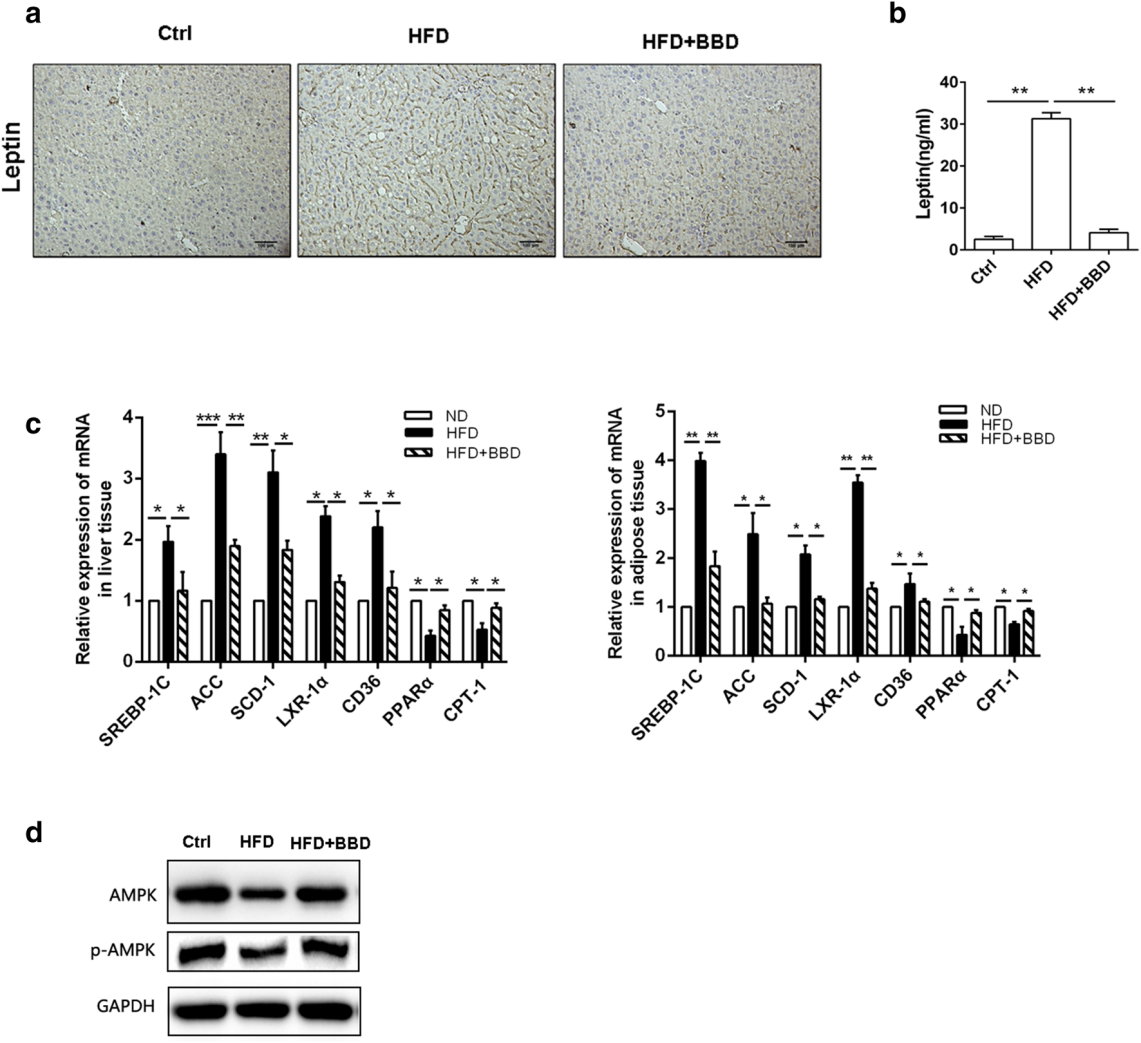
BBD activates AMPK pathway to regulate hepatic gene expression involved in lipid metabolism**. a** Concentration of Leptin in portal vein serum was detected by Leptin Elisa test kit. **b** Representative immunohistochemistry staining for leptin in liver tissues of each group. **c** The expression of hepatic genes (SREBP1-c, ACC, SCD-1, LXRα, CD36, CPT-1 and PPARα) involved in lipogenesis or β-oxidation in liver and adipose tissues of different groups. **d** Western blot was used to detect the expression of AMPK and p-AMPK in livers of each group. The data were shown as mean ± SD. n  =  7 for all grou ps. *p < 0.05,**p < 0.01

With the accumulation of fat pad in the body, adipose tissue will release leptin to suppress the produce of fat [9–11]. The leptin level in serum and liver tissue of in HFD group mice was significantly elevated, while BBD treatment group could suppress leptin expression (Fig. 4a, b). To explore the molecular mechanisms of BBD regulates hepatic fat accumulation on high fat diet induced mice, we detected the expression of genes involved in lipogenesis (sterol regulatory element-binding protein 1c (SREBP1-c), acetyl coenzyme A-carboxylase (ACC), stearoyl-CoA desaturase-1 (SCD-1), liver X receptor alpha (LXRα) and fatty acid translocase (FAT/CD36)) in the liver and adipose tissues by RT-PCR. It was showed that the expression of SREBP1-c, ACC, SCD-1, LXRα and CD36 was significantly higher in HFD-fed mice than in the normal diet mice, but returned to basal levels in the BBD administrated mice (Fig. 4c). The gene expression related to ß-oxidation (PPAR-α, CPT-1) was down-regulated in HFD-fed mice and returned to basal levels in the BBD administrated mice (Fig. 4c). BBD moderately regulated the expression of these genes of BBD + HFD group compared with untreated HFD group, suggested that protective effects of BBD on HFD-induced obesity and NAFLD may be attributed to regulating the expression of these genes related with lipid metabolism.

Previous study had showed that the activation of AMPK signaling is involved in hepatic lipid metabolism by inhibiting the activity of SREBP-1c, ACC and others [12]. To further confirm whether AMPK is involved in the regulation effects of BBD against increased HFD-induced lipid synthesis, we detected the expression of AMPK and p-AMPK in livers. We found the expression of p-AMPK was decreased in HFD group (Fig. 4d), which indicated AMPK pathway were inactivation. Meanwhile, BBD increased the expression of p-AMPK compared with untreated HFD group (Fig. 4d). These data suggest that BBD regulated lipogenesis by activating AMPK pathway.

## Discussion

Babaodan (BBD) functions mainly by clearing heat and resolving dampness, removing blood stasis, dissipating mass and relieving pain [7]. Our former study indicated that BBD ameliorated hepatic fibrosis through inhibiting activation of hepatic stellate cell and further protected liver from injury [8]. In the present study, we observed that BBD attenuates HFD-induced obesity and NAFLD. It is showed that the circulating non-esterified fatty acids pool plays an important role in the pathogenesis of NAFLD and especially the overproduction of fatty acids in adipose tissues that flow to the liver [13]. We observed the BBD treatment ameliorated the body weights and weights of liver, kidney and inguinal subcutaneous fat in mice fed high fat diet. Meanwhile, BBD significantly decreased the fat weight and adipocyte size compared with HFD mice. In addition, BBD decreased both the serum levels of glucose, TG, HDL-C and LDL-C and hepatic TG, TC in BBD group. These results suggest that BBD alleviated the progression of obesity and NAFLD.

Adipose tissue is not only consisting of adipose cells, but also including macrophages closely related to inflammation. Previous studies had reported that obesity could increase local inflammation of fat and systemic inflammation, while weight loss reduced the systemic inflammation [14, 15]. Hotamisligil et al. [16] has pointed out that the nature of obesity is a systemic chronic low inflammation induced by various inflammatory factors. According to a study, adipose tissue macrophages increase from ~ 10% of white adipose tissue cellularity in lean animals to up to 50–60% in the obese [17, 18]. To investigate whether the relive effect of BBD could alleviate inflammation, we investigated the change of inflammation. Macrophages are major inflammatory and immune effector cells that play crucial roles in producing pro-inflammatory cytokines, such as tumor necrosis factor α (TNF-α) and interleukin 6 (IL-6) [19, 20]. In this study, we found BBD administration could suppress the expression of TNF-α and IL-6. Thus, BBD ameliorated obesity-induced liver inflammation, which may be one of the reasons for alleviating liver injury in NAFLD.

Leptin is an adipocyte-derived cytokine that suppresses appetite and increases energy expenditure [21–23]. With fat accumulation in NAFLD patients, adipose tissue will release leptin. It has reported the level of leptin may represent the lipid content present in the body and increases the glucose metabolism [9–11]. Our results showed that leptin was significantly higher in mice fed with HFD than in the normal diet mice. BBD administration could restore the expression of leptin to normal level. These indicated BBD significantly reduced leptin levels in HFD-induced obese mice.

Hepatic de novo lipogenesis plays an important role in the development of NAFLD. The main pathway that regulates the initiation of fatty acid metabolism in the liver involves stimulation of SREBP-1c expression, a critical factor involved in hepatic lipid synthesis. The SREBP-1c promoter contains LXR response elements, which plays a key role in the activation of SREBP-1c transcription [24]. Mature SREBP-1c (the active form) translocates to the nucleus and promotes fatty acid biosynthesis via upregulating the expression of lipogenesis-related genes, including ACC and SCD-1 [25]. ACC active will produce malonyl-CoA blocking the build of new fatty acids and inhibiting the transfer of the fatty acyl group from acyl CoA to carnitine with carnitine acyltransferase [26]. SCD-1 is responsible for fatty acid desaturation [27, 28]. CD36 also known as fatty acid translocase, contributes to intracellular lipid accumulation [29, 30]. We confirmed the genes of SREBP1-c, ACC, SCD-1, LXRα and CD36 were up-regulation expression in HFD-fed mice than in the normal diet mice. And BBD treatment decreased the mRNA expression of these genes related to lipogenesis. PPAR-α is considered the primary factor regulating the β-oxidation of fatty acids, which limits the storage of liver fat [31]. Carnitine palmitoyl transferase enzyme 1 (CPT-1) is one of the activators of PPAR-α expressed, which promotes the uptake of fat and mitochondrial fatty acid oxidation [32]. Our data showed BBD up-regulated the expression of β-oxidation-related genes of BBD + HFD group compared with untreated HFD group. These data indicated that BBD could attenuate de novo lipogenesis and accelerate the lipid metabolism, reducing hepatic lipid accumulation.

Previous study had showed that AMPK (adenosine monophosphate-activated protein kinase) signaling regulates lipid formation and promotes glucose uptake and fatty acid oxidation in adipose tissues [33]. On the one hand, the activation of AMPK phosphorylation decreases lipid synthesis by modulating the activities of various enzymes [11, 34]. On the other, hepatic AMPK would switch off SREBP-1c and increase inactivation of ACC and others [12, 35, 36]. Our results showed the expression of AMPK and p-AMPK were decreased in mouse of HFD group, suggested that the expression of p-AMPK in liver could be reduced by long term high fat diet. BBD increased the expression of AMPK and p-AMPK compared with untreated HFD group, which suggested BBD alleviated hepatic lipogenesis by activating AMPK pathway.

## Conclusions

In summary, to the best of our knowledge this study is the first to find BBD supplementation protects against high fat diet-induced fat accumulation and non-alcoholic fatty liver disease by activation of AMPK pathway.

## Data Availability

The datasets used and/or analyzed during the current study available from the corresponding author on reasonable request.

## Abbreviations

BBD: Babaodan
NAFLD: non-alcoholic fatty liver disease
HFD: the high-fat diet
TC: total cholesterol
TG: triglyceride
HDL-C: high density lipoprotein cholesterol
LDL-C: low density lipoprotein cholesterol
ALT: alanine aminotransferase
AST: aspartate aminotransferase
LFD: low-fat diet
SREBP1-c: sterol regulatory element-binding protein 1c
ACC: acetyl coenzyme A-carboxylase
SCD-1: stearoyl-CoA desaturase-1
LXRα: liver X receptor alpha
AMPK: adenosine monophosphate-activated protein kinase
CPT-1: carnitine palmitoyl transferase enzyme 1
PPAR-α: peroxisome proliferator-activated receptor alpha
